# Successful Informal Help-Seeking and Resolution of Elder Family Financial Exploitation: A Case Study

**DOI:** 10.1093/geroni/igaa057.3217

**Published:** 2020-12-16

**Authors:** Tina Kilaberia, Marlene Stum

**Affiliations:** 1 UC Davis Health, Sacramento, California, United States; 2 University of Minnesota, Twin Cities, Saint Paul, Minnesota, United States

## Abstract

Effective interventions are needed to address elder family financial exploitation (EFFE), one of the most prevalent types of elder abuse globally. This poster examines the unique and critical help-seeking role informal family support can play when faced with EFFE. We present a holistic case study that offers an understanding of one family’s successful help-seeking and resolution of the EFFE situation. The family drew on internal family and community supports and did not seek formal elder abuse services. The case stood out as unique relative to 23 family’s help-seeking attempts in a larger study of the meaning and experience of EFFE from the perspective of concerned family members (non-abusing/non-victims). The case summary and analysis are based on an in-depth interview narrative reflecting the subjective experience of a concerned family member who was directly involved in the EFFE situation (in-law relative to both the older victim and the perpetrator). Study findings reveal 5 interwoven themes related to help-seeking processes and outcomes: 1) honoring the victim’s wishes, 2) providing support and accountability for perpetrator, 3) restoring family relationships and functioning, 4) maintaining internal (family-based) control, and 5) engaging in family problem solving processes. The family’s help-seeking demonstrated three distinctive features: a) embracing their informal social support role, b) the interdependence of family members, and c) restorative justice principles. The findings raise questions about broadening the scope and continuum of EFFE intervention research and practices to recognize and support informal social intervention.

